# Correction: Comparing methods to classify admitted patients with SARS-CoV-2 as admitted for COVID-19 versus with incidental SARS-CoV-2: A cohort study

**DOI:** 10.1371/journal.pone.0320617

**Published:** 2025-03-18

**Authors:** The PLOS One Staff

**Notice of Republication**

This article was republished on February 21, 2025, to correct an error in the third author’s name is spelled incorrectly. The correct name is: Elizabeth Purssell.

The correct citation is: Hohl CM, Cragg A, Purssell E, McAlister FA, Ting DK, Scheuermeyer F, et al. (2023) Comparing methods to classify admitted patients with SARS-CoV-2 as admitted for COVID-19 versus with incidental SARS-CoV-2: A cohort study. PLoS ONE 18(9): e0291580. https://doi.org/10.1371/journal.pone.0291580

There is an error in affiliation 3 for authors Elizabeth Purssell. The correct affiliation 2 is: Emergency Department, Royal Columbian Hospital, New Westminster, British Columbia, Canada

## Supporting information

S1 File Originally published, uncorrected article. (PDF)

S2 File Republished, corrected article. (PDF)
