# Supplementary material for: Comparing methods to classify admitted patients with SARS-CoV-2 as admitted for COVID-19 versus with incidental SARS-CoV-2: A cohort study
Source: PLoS One. 2023 Sep 26;18(9):e0291580. doi: 10.1371/journal.pone.0291580 (PMC10522023; doi:10.1371/journal.pone.0291580)
Supplement: S1 Fig — The x-axis depicts the probability of being adjudicated by clinicians as an admission primarily for COVID-19 among all cases with the same primary discharge diagnosis that were abstracted from the medical record. (DOCX) [file pone.0291580.s002.docx]

**S1 Figure. Probability of most common uncertain discharge diagnoses being categorized primarily for COVID-19**

The x-axis depicts the probability of being adjudicated by clinicians as an admission primarily for COVID-19 among all cases with the same primary discharge diagnosis that were abstracted from the medical record.
