# Supplementary material for: Comparing methods to classify admitted patients with SARS-CoV-2 as admitted for COVID-19 versus with incidental SARS-CoV-2: A cohort study
Source: PLoS One. 2023 Sep 26;18(9):e0291580. doi: 10.1371/journal.pone.0291580 (PMC10522023; doi:10.1371/journal.pone.0291580)
Supplement: S1 Table — (DOCX) [file pone.0291580.s003.docx]

**S1 Table. Hospital sites and dates of consecutive data entry**

| **Site** | **Start Date** | **End Date** | **Patients Screened** | **Patients Included** |
| --- | --- | --- | --- | --- |
| Vancouver General Hospital | 19-Dec-2021 | 1-Apr-2022 | 21998 | 417 |
| Lion's Gate Hospital | 19-Dec-2021 | 1-Jun-2022 | 1029 | 133 |
| St. Paul's Hospital | 19-Dec-2021 | 1-May-2022 | 8665 | 273 |
| Mt. St. Joseph Hospital | 19-Dec-2021 | 31-May-2022 | 2740 | 73 |
| Surrey Memorial Hospital | 19-Dec-2021 | 21-Apr-2022 | 8073 | 755 |
| **Total** | | | **42505** | **1651** |
