# Supplementary material for: Comparing methods to classify admitted patients with SARS-CoV-2 as admitted for COVID-19 versus with incidental SARS-CoV-2: A cohort study
Source: PLoS One. 2023 Sep 26;18(9):e0291580. doi: 10.1371/journal.pone.0291580 (PMC10522023; doi:10.1371/journal.pone.0291580)
Supplement: S2 Table — (DOCX) [file pone.0291580.s004.docx]

**S2 Table.** **Predefined drop-down menu of COVID-19 related diagnoses allocated to the hospitalized primarily for COVID-19 category.**

| suspected case of COVID |
| --- |
| confirmed case of COVID |
| upper respiratory tract infection |
| pneumonia |
| viral pneumonia |
| cough, nyd |
| fever, nyd |
| flu-like illness |
| ARDS Adult respiratory distress syndrome |
| SOB Shortness of breath |
| FUO Fever unknown origin |
