# Supplementary material for: Comparing methods to classify admitted patients with SARS-CoV-2 as admitted for COVID-19 versus with incidental SARS-CoV-2: A cohort study
Source: PLoS One. 2023 Sep 26;18(9):e0291580. doi: 10.1371/journal.pone.0291580 (PMC10522023; doi:10.1371/journal.pone.0291580)
Supplement: S3 Table — (DOCX) [file pone.0291580.s005.docx]

**S3 Table.** **Free text diagnoses allocated to the hospitalized primarily for COVID-19 category.**

| community acquired pneumonia |
| --- |
| COVID delirium |
| COVID pneumonia |
| croup |
| hemoptysis/hematemesis in context of severe cough and vomiting with COVID infection |
| hypercapnic respiratory failure |
| hypoexemic respiratory failure |
| hypoxemic respiratory failiure |
| hypoxemic respiratory failure |
| hypoxemic respiratory failure secondary to COVID pneumonia |
| hypoxic respiratory failure |
| laryngitis |
| left upper lobe pneumonia |
| post COVID viral inflammatory syndrome |
| respiratory arrest |
| respiratory failure secondary to COVID -19 |
| suspected left-sided empyema versus pneumonia |
| viral respiratory infection, nyd |
