# Supplementary material for: Comparing methods to classify admitted patients with SARS-CoV-2 as admitted for COVID-19 versus with incidental SARS-CoV-2: A cohort study
Source: PLoS One. 2023 Sep 26;18(9):e0291580. doi: 10.1371/journal.pone.0291580 (PMC10522023; doi:10.1371/journal.pone.0291580)
Supplement: S4 Table — (DOCX) [file pone.0291580.s006.docx]

**S4 Table.** **Free text discharge diagnoses deemed ‘uncertain’ if with incidental COVID-19 or ‘for’ incidental COVID-19.**

| abdominal pain |
| --- |
| abdominal pain most likely secondary to gastritis |
| abdominal sepsis |
| acute chest syndrome |
| acute exacerbation of heart failure |
| acute kidney injury |
| acute myocarditis |
| acute on chronic compression fracture at l1 with retropulsion |
| acute on chronic diarrhea |
| acute on chronic kidney injury |
| acute on chronic lung allograft dysfunction |
| acute on chronic renal dysfunction |
| acute pulmonary embolism |
| acute renal failure |
| advanced dementia |
| advanced dementia with bpsd |
| altered level of consciousness |
| alzheimer's dementia |
| aspiration pneumonia |
| aspiration pneumonitis |
| asystolic cardiac arrest |
| bilateral leg weakness |
| bilateral parotid gland swelling |
| blood culture positive was skin contaminant. no infection |
| c6 endplate avulsion/fracture |
| cardiac arrest |
| cardiorespiratory failure |
| cerebrovascular accident |
| chronic kidney disease |
| chronic pain |
| chronic renal failure |
| colitis |
| colitis, nyd |
| complex febrile seizure |
| confusion, altered level of consciousness, nyd |
| csf pleocytosis |
| deconditioning |
| decreased level of consciousness |
| dehydration |
| delirium |
| delirium complicating previous history of vascular dementia |
| delirium nyd |
| dementia |
| dementia with bpsd |
| dementia with functional decline |
| desquamating dermatitis |
| diarrhea |
| diarrhea nyd |
| diarrhea, likely viral gastroenteritis |
| disposition planning |
| donor kidney transplant dysfunction |
| encephalitis |
| encephalopathy |
| end stage renal disease |
| end-stage parkinson’s disease |
| end-stage renal disease |
| failed renal transplant |
| failure to thrive |
| fall |
| fall and query tremor |
| fall mechanical |
| fall with facial fractures |
| fall with left thigh pain |
| fall with soft tissue injuries |
| fall, left humerus fracture |
| fall, with intracranial hemorrhage |
| falls |
| falls with vascular dementia |
| febrile neutropenia |
| fracture hip intertrochanteric closed |
| fracture patella closed |
| fracture, hip |
| fractured left distal radius |
| frailty and decreased function with poor oral intake |
| functional decline |
| functional decline, failure to thrive |
| gastroenteritis |
| gastroparesis |
| general weakness |
| general weakness nyd |
| generalized weakness |
| heart failure exacerbation |
| hemoptysis |
| hepatitis |
| high output ileostomy |
| hip fracture |
| housing |
| hyperglycemia |
| hypernatremia |
| hypoactive delirium |
| hypoglycemia |
| hyponatremia |
| hyponatremia multifactorial decreased p.o. intake |
| hypotension orthostatic |
| hypovolemia |
| hypoxic ischemic brain injury |
| idiopathic parkinson's disease |
| immobility |
| infection |
| interstitial lung disease |
| labile hyperglycemia and hypoglycemia |
| left ankle fracture |
| left distal humerus intra-articular fracture |
| left femoral neck fracture |
| left femur periprosthetic fracture |
| left hip fracture |
| left intertrochanteric hip fracture |
| left intracapsular neck of femur fracture |
| left proximal tibia fracture |
| left subcapital hip fracture |
| left wrist fracture post fall |
| low back pain |
| malaise |
| mechanical back pain |
| mechanical fall |
| medication interaction |
| mucositis |
| multi system organ failure |
| multifactorial delirium |
| multifatorial dyspnea |
| multiorgan failure |
| musculoskeletal disorders nos |
| nausea, vomiting and reduced oral intake |
| neurological disease nos |
| non-operative left sup/infer pubic rami fracture |
| obesity hypoventilation syndrome |
| occipital condyle fracture |
| olecranon fracture |
| orthostatic hypotension |
| out of hospital cardiac arrest |
| out-of-hospital cardiac arrest |
| parkinson's |
| parkinson's disease |
| pea arrest |
| pleural effusion |
| polycythemia |
| post critical care weakness |
| prerenal acute kidney injury |
| prodromal syncope -- hypovolemia |
| progressive dementia |
| progressive hypoxemic respiratory failure |
| prolonged qtc secondary to metabolic derangements |
| query diabetic gastroparesis |
| recurrent fall |
| recurrent gastroparesis |
| recurrent symptomatic hypotension nyd |
| recurrent syncope |
| refractory non-convulsive status epilepticus in the setting of post-ohca |
| refractory vasoplegic shock secondary to prolonged pea sirs |
| rehabilitation |
| renal failure |
| right ankle fracture |
| right ankle fracture, bimalleolar |
| right femoral neck fracture |
| right femur periprosthetic fracture |
| right hip fracture |
| right hip fracture, pubic ramus |
| right hip intertrochanteric fracture |
| right intertrochanteric hip fracture |
| right pleural effusion |
| right subtrochanteric fracture |
| sepsis |
| sepsis/cellulitis |
| severe acute kidney injury |
| severe hyponatremia |
| significant hyponatremia of 118 |
| starvation ketoacidosis |
| suspected fall resulting in l2-l3 fracture |
| suspected urosepsis |
| syncopal episode |
| syncope |
| syncope nos |
| syncope, nyd |
| syncope, presumed orthostatic hypotension and reflex syncope mediated |
| type 2 myocardial infarction |
| vasovagal syncope |
| vasovagal syncope secondary to hypoglycemia |
| vertigo |
| very frail |
| weakness |
| weakness and frailty |
