# Supplementary material for: Comparing methods to classify admitted patients with SARS-CoV-2 as admitted for COVID-19 versus with incidental SARS-CoV-2: A cohort study
Source: PLoS One. 2023 Sep 26;18(9):e0291580. doi: 10.1371/journal.pone.0291580 (PMC10522023; doi:10.1371/journal.pone.0291580)
Supplement: S5 Table — (DOCX) [file pone.0291580.s007.docx]

**S5 Table. Interrater agreement for classifying patients hospitalized primarily for COVID-19 and with incidental SARS-CoV-2 between research assistants and two physicians.**

| **Vancouver General Hospital** | **Rater** | | |
| --- | --- | --- | --- |
|  | *Research Assistant* | *Physician 1* | *Physician 2* |
| Hospitalized primarily for COVID | 15 | 17 | 17 |
| Hospitalized with COVID | 18 | 16 | 16 |
| **Fleiss’ Kappa: 0.91 (95%CI: 0.80, 1)** | | | |
|  | | | |
| **St. Paul’s Hospital/ Mt. St Joseph’s** | **Rater** | | |
|  | *Research Assistant* | *Physician 1* | *Physician 2* |
| Hospitalized primarily for COVID | 19 | 21 | 21 |
| Hospitalized with COVID | 17 | 15 | 15 |
| **Fleiss’ Kappa: 0.92 (95%CI: 0.81, 1)** | | | |
|  | | | |
| **Surrey Memorial Hospital** | **Rater** | | |
|  | *Research Assistant* | *Physician 1* | *Physician 2* |
| Hospitalized primarily for COVID | 12 | 16 | 16 |
| Hospitalized with COVID | 19 | 15 | 15 |
| **Fleiss’ Kappa: 0.83 (95%CI: 0.66, 0.96)** | | | |
