# Supplementary material for: Comparing methods to classify admitted patients with SARS-CoV-2 as admitted for COVID-19 versus with incidental SARS-CoV-2: A cohort study
Source: PLoS One. 2023 Sep 26;18(9):e0291580. doi: 10.1371/journal.pone.0291580 (PMC10522023; doi:10.1371/journal.pone.0291580)
Supplement: S6 Table — (DOCX) [file pone.0291580.s008.docx]

**S6 Table. Interrater agreement for classifying patients hospitalized primarily for COVID-19 and with incidental SARS-CoV-2 who had a free text diagnosis deemed to be uncertain.**

| **Lions Gate Hospital** | **Rater** | |
| --- | --- | --- |
|  | *Physician 1* | *Physician 2* |
| Hospitalized primarily For COVID | Suppressed | Suppressed |
| Hospitalized With COVID | 8 | 8 |
| **Fleiss’ Kappa: 1.00 (95%CI: 1.00, 1.00)** | | |
|  | | |
| **St. Paul’s Hospital/ Mt. St Joseph’s** | **Rater** | |
|  | *Physician 1* | *Physician 2* |
| Hospitalized primarily For COVID | 26 | 26 |
| Hospitalized With COVID | 22 | 22 |
| **Fleiss’ Kappa: 1.00 (95%CI: 1.00, 1.00)** | | |
|  | | |
| **Surrey Memorial Hospital** | **Rater** | |
|  | *Physician 1* | *Physician 2* |
| Hospitalized primarily For COVID | 61 | 64 |
| Hospitalized With COVID | 105 | 102 |
| **Fleiss’ Kappa: 0.91 (95%CI: 0.85, 0.98)** | | |
|  | | |
| **Vancouver General Hospital** | **Rater** | |
|  | *Physician 1* | *Physician 2* |
| Hospitalized primarily For COVID | 22 | 21 |
| Hospitalized With COVID | 42 | 43 |
| **Fleiss’ Kappa: 0.90 (95%CI: 0.78, 1.00)** | | |
