# Supplementary material for: Comparing methods to classify admitted patients with SARS-CoV-2 as admitted for COVID-19 versus with incidental SARS-CoV-2: A cohort study
Source: PLoS One. 2023 Sep 26;18(9):e0291580. doi: 10.1371/journal.pone.0291580 (PMC10522023; doi:10.1371/journal.pone.0291580)
Supplement: S7 Table — (DOCX) [file pone.0291580.s009.docx]

**S7 Table**. **The most common primary discharge diagnoses among discordant cases when comparing the clinical decision and the CDC methods of classification**

| **Primary discharge diagnoses*** | **Type 1 Disagreement**  **(Clinical Decision=**  **Primarily for COVID)**  (n=147) | **Type 2 Disagreement**  **(CDC Algorithm=**  **Primarily for COVID)**  **(**n=15**)** |
| --- | --- | --- |
| Pneumonia | 24 | 0 |
| Failure to thrive | 20 | 0 |
| Altered level of consciousness | 18 | 0 |
| Fall | 10 | 0 |
| Hyponatremia | 10 | 0 |
| Kidney injury | 10 | 0 |
| Sepsis | <5 | 7 |
| Pulmonary embolism | 0 | 6 |
| Upper respiratory tract infection | 6 | 0 |
| Fever | 5 | 0 |
