# Supplementary material for: Comparing methods to classify admitted patients with SARS-CoV-2 as admitted for COVID-19 versus with incidental SARS-CoV-2: A cohort study
Source: PLoS One. 2023 Sep 26;18(9):e0291580. doi: 10.1371/journal.pone.0291580 (PMC10522023; doi:10.1371/journal.pone.0291580)
Supplement: S8 Table — (DOCX) [file pone.0291580.s010.docx]

**S8 Table**. **The most common primary discharge diagnoses among discordant cases when comparing the clinical decision and the Massachusetts method of classification**

| **Primary discharge diagnoses*** | **Type 1 Disagreement**  **(Clinical Decision=**  **Primarily for COVID)**  (n=339) | **Type 2 Disagreement**  **(Massachusetts Method=**  **Primarily for COVID)**  **(**n=170**)** |
| --- | --- | --- |
| COVID-19 | 229 | 0 |
| Cancer | 0 | 23 |
| Failure to thrive | 17 | 6 |
| Pneumonia | 19 | 0 |
| Sepsis | 0 | 18 |
| Altered level of consciousness | 14 | <5 |
| Fall | 7 | 8 |
| Kidney injury | 8 | <5 |
| Heart failure | 0 | 9 |
| Hyponatremia | 9 | 0 |
