# Supplementary material for: Comparing methods to classify admitted patients with SARS-CoV-2 as admitted for COVID-19 versus with incidental SARS-CoV-2: A cohort study
Source: PLoS One. 2023 Sep 26;18(9):e0291580. doi: 10.1371/journal.pone.0291580 (PMC10522023; doi:10.1371/journal.pone.0291580)
Supplement: S9 Table — OR = odds ratio; ICU = intensive care unit; CI = confidence interval. a active malignant neoplasm, transplant recipient, moderate/severe liver disease. Hospital site was included as a fixed effect in this model. For simplicity, site estimates were excluded from the table. (DOCX) [file pone.0291580.s011.docx]

**S9 Table. Factors associated with ventilation, critical care admission or mortality among 1,651 SARS-CoV-2 positive patients, according to clinician decision.**

| **Risk Factor** | | **Outcome** | | | | | |
| --- | --- | --- | --- | --- | --- | --- | --- |
|  |  | **Mechanical**  **Ventilation**  (n=1651) | | **ICU Admission**  (n=1651) | | **Mortality**  (n=1651) | |
|  |  | **OR** | **95%CI** | **OR** | **95%CI** | **OR** | **95%CI** |
| Age (per 10-years) |  | 0.88 | 0.78, 1.01 | 0.88 | 0.82, 0.95 | 1.50 | 1.33, 1.70 |
| Sex |  |  |  |  |  |  |  |
|  | *Female* | — | — | — | — | — | — |
|  | *Male* | 1.94 | 1.08, 3.47 | 1.71 | 1.21, 2.41 | 1.46 | 1.01, 2.09 |
| Secondary immunodeficiency^a^ |  |  |  |  |  |  |  |
|  | *No* | — | — | — | — | — | — |
|  | *Yes* | 0.94 | 0.47, 1.90 | 1.36 | 0.91, 2.03 | 1.91 | 1.29, 2.84 |
| Obesity |  |  |  |  |  |  |  |
|  | *No* | — | — | — | — | — | — |
|  | *Yes* | 1.60 | 0.60, 4.26 | 1.63 | 0.87, 3.06 | 1.26 | 0.58, 2.75 |
| Omicron Variant |  |  |  |  |  |  |  |
|  | *BA.1* | — | — | — | — | — | — |
|  | T*ransition* | 0.96 | 0.52, 1.79 | 1.19 | 0.82, 1.74 | 1.00 | 0.66, 1.51 |
|  | *BA.2* | 0.52 | 0.15, 1.76 | 0.56 | 0.27, 1.15 | 0.39 | 0.17, 0.94 |
| Clinician classification of for vs with COVID-19 |  |  |  |  |  |  |  |
|  | *With COVID* | — | — | — | — | — | — |
|  | Primarily *For COVID* | 1.18 | 0.69, 2.03 | 1.86 | 1.31, 2.63 | 1.49 | 1.03, 2.16 |
| Illicit substance use |  |  |  |  |  |  |  |
|  | *No* | — | — | — | — | — | — |
|  | *Yes* | 2.01 | 1.06, 3.78 | 1.24 | 0.79, 1.95 | 1.02 | 0.49, 2.11 |
| Any vaccine dose received 7-days prior to ED visit |  |  |  |  |  |  |  |
|  | *No* | — | — | — | — | — | — |
|  | *Yes* | 0.55 | 0.31, 0.96 | 0.51 | 0.35, 0.74 | 0.89 | 0.56, 1.40 |

OR=odds ratio; ICU=intensive care unit; CI=confidence interval

^a^active malignant neoplasm, transplant recipient, moderate/severe liver disease

Hospital site was included as a fixed effect in this model. For simplicity, site estimates were excluded from the table.
