# Supplementary material for: Comparing methods to classify admitted patients with SARS-CoV-2 as admitted for COVID-19 versus with incidental SARS-CoV-2: A cohort study
Source: PLoS One. 2023 Sep 26;18(9):e0291580. doi: 10.1371/journal.pone.0291580 (PMC10522023; doi:10.1371/journal.pone.0291580)
Supplement: S10 Table — OR = odds ratio; ICU = intensive care unit; CI = confidence interval. a active malignant neoplasm, transplant recipient, moderate/severe liver disease. Hospital site was included as a fixed effect in this model. For simplicity, site estimates were excluded from the table. (DOCX) [file pone.0291580.s012.docx]

**S10 Table. Factors associated with ventilation, critical care admission or mortality among 1,651 SARS-CoV-2 positive patients, according to the CDC admission classification.**

| **Risk Factor** | | **Outcome** | | | | | |
| --- | --- | --- | --- | --- | --- | --- | --- |
|  |  | **Mechanical Ventilation**  (n=1651) | | **ICU Admission**  (n=1651) | | **Mortality**  (n=1651) | |
|  |  | **OR** | **95%CI** | **OR** | **95%CI** | **OR** | **95%CI** |
| Age (per 10-years) |  | 0.89 | 0.78, 1.01 | 0.89 | 0.82, 0.96 | 1.50 | 1.33, 1.70 |
| Sex |  |  |  |  |  |  |  |
|  | *Female* | — | — | — | — | — | — |
|  | *Male* | 1.94 | 1.09, 3.48 | 1.71 | 1.21, 2.42 | 1.45 | 1.00, 2.08 |
| Secondary immunodeficiency^a^ |  |  |  |  |  |  |  |
|  | *No* | — | — | — | — | — | — |
|  | *Yes* | 0.96 | 0.48, 1.93 | 1.38 | 0.92, 2.04 | 1.91 | 1.29, 2.84 |
| Obesity |  |  |  |  |  |  |  |
|  | *No* | — | — | — | — | — | — |
|  | *Yes* | 1.61 | 0.61, 4.29 | 1.50 | 0.80, 2.81 | 1.17 | 0.54, 2.56 |
| Omicron variant |  |  |  |  |  |  |  |
|  | *BA.1* | — | — | — | — | — | — |
|  | T*ransition* | 0.95 | 0.51, 1.76 | 1.20 | 0.83, 1.76 | 1.01 | 0.67, 1.52 |
|  | *BA.2* | 0.52 | 0.15, 1.76 | 0.56 | 0.27, 1.14 | 0.38 | 0.16, 0.91 |
| CDC classification of for vs with COVID-19 |  |  |  |  |  |  |  |
|  | *With COVID* | — | — | — | — | — | — |
|  | Primarily *For COVID* | 1.05 | 0.61, 1.82 | 2.24 | 1.59, 3.16 | 1.85 | 1.29, 2.65 |
| Illicit substance use |  |  |  |  |  |  |  |
|  | *No* | — | — | — | — | — | — |
|  | *Yes* | 1.97 | 1.04, 3.70 | 1.27 | 0.81, 2.00 | 1.06 | 0.51, 2.21 |
| Any vaccine dose received 7-days prior to ED visit |  |  |  |  |  |  |  |
|  | *No* | — | — | — | — | — | — |
|  | *Yes* | 0.54 | 0.30, 0.95 | 0.53 | 0.37, 0.77 | 0.94 | 0.59, 1.48 |

OR=odds ratio; ICU=intensive care unit; CI=confidence interval

^a^ active malignant neoplasm, transplant recipient, moderate/severe liver disease

Hospital site was included as a fixed effect in this model. For simplicity, site estimates were excluded from the table.
