# Supplementary material for: Comparing methods to classify admitted patients with SARS-CoV-2 as admitted for COVID-19 versus with incidental SARS-CoV-2: A cohort study
Source: PLoS One. 2023 Sep 26;18(9):e0291580. doi: 10.1371/journal.pone.0291580 (PMC10522023; doi:10.1371/journal.pone.0291580)
Supplement: S11 Table — OR = odds ratio; ICU = intensive care unit; CI = confidence interval. a active malignant neoplasm, transplant recipient, moderate/severe liver disease. Hospital site was included as a fixed effect in this model. For simplicity, site estimates were excluded from the table. (DOCX) [file pone.0291580.s013.docx]

**S11 Table. Factors associated with ventilation, critical care admission or mortality among 1,651 SARS-CoV-2 positive patients, according to the Massachusetts admission classification.**

| **Risk Factor** | | **Outcome** | | | | | |
| --- | --- | --- | --- | --- | --- | --- | --- |
|  |  | **Mechanical Ventilation**  (n=1651) | | **ICU Admission**  (n=1651) | | **Mortality**  (n=1651) | |
|  |  | **OR** | **95%CI** | **OR** | **95%CI** | **OR** | **95%CI** |
| Age (per 10-years) |  | 0.84 | 0.74, 0.96 | 0.84 | 0.77, 0.91 | 1.50 | 1.33, 1.70 |
| Sex |  |  |  |  |  |  |  |
|  | *Female* | — | — | — | — | — | — |
|  | *Male* | 1.72 | 0.96, 3.11 | 1.54 | 1.08, 2.20 | 1.33 | 0.92, 1.93 |
| Secondary immunodeficiency^a^ |  |  |  |  |  |  |  |
|  | *No* | — | — | — | — | — | — |
|  | *Yes* | 0.84 | 0.41, 1.69 | 1.29 | 0.86, 1.93 | 1.89 | 1.26, 2.83 |
| Obesity |  |  |  |  |  |  |  |
|  | *No* | — | — | — | — | — | — |
|  | *Yes* | 1.58 | 0.58, 4.26 | 1.62 | 0.85, 3.09 | 1.16 | 0.52, 2.56 |
| Omicron variant |  |  |  |  |  |  |  |
|  | *BA.1* | — | — | — | — | — | — |
|  | T*ransition* | 1.00 | 0.53, 1.87 | 1.24 | 0.84, 1.83 | 1.08 | 0.70, 1.64 |
|  | *BA.2* | 0.60 | 0.17, 2.04 | 0.62 | 0.30, 1.29 | 0.41 | 0.17, 0.98 |
| Massachusetts classification of for vs with COVID |  |  |  |  |  |  |  |
|  | *With SARS-CoV-2* | — | — | — | — | — | — |
|  | Primarily *For COVID* | 3.85 | 2.17, 6.85 | 5.16 | 3.55, 7.49 | 4.72 | 3.15, 7.07 |
| Illicit substance use |  |  |  |  |  |  |  |
|  | *No* | — | — | — | — | — | — |
|  | *Yes* | 2.44 | 1.29, 4.62 | 1.44 | 0.90, 2.29 | 1.15 | 0.55, 2.41 |
| Any vaccine dose received 7-days prior to ED visit |  |  |  |  |  |  |  |
|  | *No* | — | — | — | — | — | — |
|  | *Yes* | 0.61 | 0.35, 1.09 | 0.57 | 0.39, 0.83 | 1.08 | 0.68, 1.72 |

OR=odds ratio; ICU=intensive care unit; CI=confidence interval

^a^ active malignant neoplasm, transplant recipient, moderate/severe liver disease

Hospital site was included as a fixed effect in this model. For simplicity, site estimates were excluded from the table.
