# Supplementary material for: Correction: Comparing methods to classify admitted patients with SARS-CoV-2 as admitted for COVID-19 versus with incidental SARS-CoV-2: A cohort study
Source: PLoS One. 2025 Mar 18;20(3):e0320617. doi: 10.1371/journal.pone.0320617 (PMC11918412; doi:10.1371/journal.pone.0320617)
Supplement: S1 File — (PDF) [file pone.0320617.s001.pdf]

PLOS ONE

RESEARCH ARTICLE

Comparing methods to classify admitted patients with SARS-CoV-2 as admitted for COVID-19 versus with incidental SARS-CoV-2: A cohort study

Corinne M. Hohl[1,2]*, Amber Cragg[1], Elizabeth Purssel[1,3], Finlay A. McAlister[4,5], Daniel K. Ting[1,2], Frank Scheuermeyer[1,6], Maja Stachura[1,7], Lars Grant[8,9], John Taylor[1], Josephine Kanu[1], Jeffrey P. Hau[1], Ivy Cheng[10,11], Clare L. Atzema[10,12,13], Rajan Bola[1], Laurie J. Morrison[11,13], Megan Landes[11,14], Jeffrey J. Perry[15,16], Rhonda J. Rosychuk[17], the Canadian COVID-19 Emergency Department Rapid Response Network (CCEDRRN) investigators for the Network of Canadian Emergency Researchers, the Canadian Critical Care Trials Group¶

1 Department of Emergency Medicine, University of British Columbia, Vancouver, British Columbia, Canada, **2** Emergency Department, Vancouver General Hospital, Vancouver, British Columbia, Canada, **3** Emergency Department, Surrey Memorial Hospital, Surrey, British Columbia, Canada, **4** Division of General Internal Medicine, University of Alberta, Edmonton, Alberta, Canada, **5** Alberta Strategy for Patient Oriented Research Support Unit, Edmonton, Alberta, Canada, **6** Emergency Department, St. Paul's & Mount Saint Joseph Hospitals, Vancouver, British Columbia, Canada, **7** Emergency Department, Lions Gate Hospital, North Vancouver, British Columbia, Canada, **8** Department of Emergency Medicine, McGill University, Montreal, Quebec, Canada, **9** Lady Davis Institute for Medical Research, Montreal, Quebec, Canada, **10** Sunnybrook Research Institute, Sunnybrook Health Sciences Centre, Toronto, Ontario, Canada, **11** Division of Emergency Medicine, Department of Medicine, University of Toronto, Toronto, Ontario, Canada, **12** Institute for Clinical Evaluative Sciences, Toronto, Ontario, Canada, **13** Department of Emergency Services, Sunnybrook Health Sciences Centre, Toronto, Ontario, Canada, **14** University Health Network, Toronto, Ontario, Canada, **15** Department of Emergency Medicine, University of Ottawa, Ottawa, Ontario, Canada, **16** Ottawa Hospital Research Institute, Ottawa, Ontario, Canada, **17** Department of Pediatrics, University of Alberta, Edmonton, Alberta, Canada

¶ Membership of the Canadian COVID-19 Emergency Department Rapid Response Network (CCEDRRN) investigators for the Network of Canadian Emergency Researchers and the Canadian Critical Care Trials Group is provided in the S1 File.
* corinne.hohl@ubc.ca

Check for updates

OPEN ACCESS

Citation: Hohl CM, Cragg A, Purssel E, McAlister FA, Ting DK, Scheuermeyer F, et al. (2023) Comparing methods to classify admitted patients with SARS-CoV-2 as admitted for COVID-19 versus with incidental SARS-CoV-2: A cohort study. PLoS ONE 18(9): e0291580. https://doi.org/10.1371/journal.pone.0291580

Editor: Jung Yeon Heo, Ajou University School of Medicine, REPUBLIC OF KOREA

Received: April 12, 2023

Accepted: August 31, 2023

Published: September 26, 2023

Copyright: © 2023 Hohl et al. This is an open access article distributed under the terms of the Creative Commons Attribution License, which permits unrestricted use, distribution, and reproduction in any medium, provided the original author and source are credited.

Data Availability Statement: Our clinical data are collected with a waiver of informed consent. Authors do not have permission to share these data. Data access is governed by the CCEDRRN Protocol Review and Publications Committee and Data Access and Management Committee (contact via amber.cragg@ubc.ca) for researchers who meet the criteria for access to confidential data.

Funding: Our network is funded by the Canadian Institutes of Health Research (447679, 464947,

Abstract

Introduction

Not all patients with severe acute respiratory syndrome coronavirus 2 (SARS-CoV-2) infection develop symptomatic coronavirus disease 2019 (COVID-19), making it challenging to assess the burden of COVID-19-related hospitalizations and mortality. We aimed to determine the proportion, resource utilization, and outcomes of SARS-CoV-2 positive patients admitted for COVID-19, and assess the impact of using the Center for Disease Control's (CDC) discharge diagnosis-based algorithm and the Massachusetts state department's drug administration-based classification system on identifying admissions for COVID-19.

Methods

In this retrospective cohort study, we enrolled consecutive SARS-CoV-2 positive patients admitted to one of five hospitals in British Columbia between December 19, 2021 and May

and 466880), Ontario Ministry of Colleges and Universities (C-655-2129), Saskatchewan Health Research Foundation (5357), Genome BC (COV024 and VAC007), Fondation du CHU de Québec (Octroi No. 4007), and the Public Health Agency of Canada/COVID-19 Immunity Task Force (2122-HQ-000054) provided peer-reviewed funding. The BC Academic Health Science Network and BioTalent Canada provided non-peer reviewed funding. These organizations are not-for-profit, and had no role in study conduct, analysis, or manuscript preparation.

Competing interests: Drs. Perry and Atzema have peer reviewed mid-career salary support awards from the Heart and Stroke Foundation of Ontario. Dr. Hohl is supported by a Michael Smith Foundation Health Professional Investigator Award. This does not alter our adherence to PLOS ONE policies on sharing data and materials.

31,2022. We completed medical record reviews, and classified hospitalizations as being primarily for COVID-19 or with incidental SARS-CoV-2 infection. We applied the CDC algorithm and the Massachusetts classification to estimate the difference in hospital days, intensive care unit (ICU) days and in-hospital mortality and calculated sensitivity and specificity.

Results

Of 42,505 Emergency Department patients, 1,651 were admitted and tested positive for SARS-CoV-2, with 858 (52.0%, 95% CI 49.6–54.4) admitted for COVID-19. Patients hospitalized for COVID-19 required ICU admission (14.0% versus 8.2%, p<0.001) and died (12.6% versus 6.4%, p<0.001) more frequently compared with patients with incidental SARS-CoV-2. Compared to case classification by clinicians, the CDC algorithm had a sensitivity of 82.9% (711/858, 95% CI 80.3%, 85.4%) and specificity of 98.1% (778/793, 95% CI 97.2%, 99.1%) for COVID-19-related admissions and underestimated COVID-19 attributable hospital days. The Massachusetts classification had a sensitivity of 60.5% (519/858, 95% CI 57.2%, 63.8%) and specificity of 78.6% (623/793, 95% CI 75.7%, 81.4%) for COVID-19-related admissions, underestimating total number of hospital and ICU bed days while overestimating COVID-19-related intubations, ICU admissions, and deaths.

Conclusion

Half of SARS-CoV-2 hospitalizations were for COVID-19 during the Omicron wave. The CDC algorithm was more specific and sensitive than the Massachusetts classification, but underestimated the burden of COVID-19 admissions.

Trial registration

Clinicaltrials.gov, NCT04702945.

Introduction

During the first two years of the coronavirus disease 2019 (COVID-19) pandemic, widespread molecular testing for severe acute respiratory syndrome coronavirus 2 (SARS-CoV-2) enabled comprehensive case identification for isolation and contract tracing, while generating health data to evaluate risk factors, prognosis, vaccine and therapeutic effectiveness, and to plan health system resources. However, by the end of 2021, exponential growth in SARS-CoV-2 cases overwhelmed community-based testing, which, combined with increases in mild illness and more frequent self-testing at home rendered reported outpatient COVID-19 case counts incomplete [1]. In 2022, diagnosed and documented COVID-19 cases vastly underrepresented actual cases, such that new metrics were needed for surveillance, research, and health system planning [2].

Diagnostic testing in hospitals remained widely available and ensured complete or near complete case ascertainment among hospitalized patients until the end of 2021 [3, 4]. As the Omicron wave progressed, clinicians witnessed more and more SARS-CoV-2 positive patients being hospitalized with symptoms *not* attributable to COVID-19 (e.g., ectopic pregnancy) [8]. Algorithms based on provider-assigned discharge diagnoses and in-hospital drug administration (e.g., dexamethasone or remdesivir) were developed to try and distinguish between hospitalizations primarily for COVID-19 from those with incidental SARS-CoV-2 infection, but

none have been validated, and they omit atypical presentations (e.g., hyponatremia from COVID-19 related syndrome of inappropriate antidiuretic hormone secretion) which are recognized presentations of COVID-19 [3, 5–7]. As a result, discharge diagnosis- or drug administration-based algorithms may misclassify COVID-19 admissions, which could impact surveillance, research, and underestimate the resources needed to look after COVID-19 patients if used for health system planning [5].

Our main objective was to assess the proportion of SARS-CoV-2 positive patients admitted for COVID-19 during the Omicron wave, and assess the impact of classifying SARS-CoV-2 admissions using a discharge-diagnosis based algorithm developed by the Center for Disease Control (CDC) currently used to monitor temporal trends [6] and a drug administration-based classification scheme proposed by Fillmore et al. currently used by the Massachusetts state department to estimate the true burden of disease for COVID-19 in their hospitals [3, 8]. Secondary objectives included assessing risk factors, outcomes, and resource utilization for COVID-19 admissions versus those with incidental SARS-CoV-2.

Methods

Design and setting

This multicenter retrospective cohort study was a planned analysis of patients enrolled in the Canadian COVID-19 Emergency Department Rapid Response Network (CCEDRRN), a national collaboration that harmonized data collection on consecutive SARS-CoV-2 tested patients presenting to Emergency Departments across eight Canadian provinces [9–12]. We included data from five participating urban acute care hospitals in British Columbia (BC) that were able to provide timely access to medical records for detailed chart review (S1 Table).

We used publicly available genomic sequencing data from BC's Public Health laboratory to define the study period. We defined variant dominance as a period of time when ≥90% of sequenced samples were attributed to one variant or subvariant [13]. Omicron BA.1 became the dominant variant at study sites on December 19, 2021, which marked the beginning of the study period. BA.1 remained dominant until February 12, 2022, followed by a transition period, after which BA.2 became dominant starting on April 3, 2022. The study period ended between April 1 and June 1, 2022 depending on the study site (S1 Table).

Participants

All participating hospitals had mandatory SARS-CoV-2 testing protocols in place for all patients requiring admission during the study period, allowing us to capture a complete sample of SARS-CoV-2 positive patients. We included all consecutive eligible Emergency Department patients when first hospitalized with a positive SARS-CoV-2 nucleic acid amplification test from a specimen collected 14 days or less prior to hospital arrival (accounting for the natural progression from infection to severe disease) or during the first five days of admission (to account for the incubation period and resulting initial false negative tests) (S1 Table) [14, 15]. Patients were followed-up via medical record review after in-hospital death or hospital discharge. We excluded patients who were discharged from the Emergency Department, and those remaining hospitalized after August 2, 2022, as we were unable to ascertain their outcomes.

Definitions

No explicit clinical definitions have been published that define hospitalizations primarily for COVID-19 versus with incidental SARS-CoV-2 infection. Thus, we developed a clinical

definition to define hospitalizations as being primarily for COVID-19 if patients tested SARS--CoV-2 positive, were hospitalized primarily due to COVID-19 attributable signs and symptoms based on the World Health Organization COVID-19 core case report form [16], and no plausible alternative diagnosis was made to explain their signs and symptoms (e.g., bacteremia to explain sepsis, hydrochlorothiazide to explain hyponatremia). We defined hospitalizations with incidental SARS-CoV-2 infection as hospitalizations in which patients tested SARS-CoV-2 positive, were hospitalized with signs and symptoms other than those attributable to COVID-19 or had an alternative diagnosis that better explained their signs and symptoms or were diagnosed with an exacerbation of a chronic illness that may or may not have been causally related to COVID-19.

We defined severe COVID-19 according to the World Health Organization age-based criteria [17]. For adults, this included an oxygen saturation of $<90\%$ on room air, a respiratory rate >30 breaths per minute, or signs of severe respiratory distress documented in the medical record.

We applied the CDC algorithm, which has not previously been validated, proposed by researchers to identify hospitalizations for COVID-19 using provider-assigned diagnoses and treatments [6]. The CDC algorithm categorizes hospitalizations as being for COVID-19 if the primary discharge diagnosis was COVID-19, or if a patient had a secondary discharge diagnosis of COVID-19 and they were either treated with remdesivir or their primary diagnosis was sepsis, pulmonary embolism, acute respiratory failure, or pneumonia.

We also applied the drug administration-based method suggested by Fillmore et al. currently being used by the Massachusetts state department to classify hospitalizations as primarily for COVID-19 versus with incidental SARS-CoV-2 [3, 8]. The Massachusetts method categorizes hospitalizations as being for COVID-19 if the patient received dexamethasone at any time during their hospital visit.

Data sources

Trained research assistants abstracted data from paper-based and electronic medical records, including demographic variables, housing situation, arrival mode and acuity, infection risk, co-morbidities, code status, substance use, Canadian Triage and Acuity Scale (CTAS) score [18], vaccination status, treatments received, laboratory tests, diagnostic imaging, vital signs, presenting symptoms, oxygen and respiratory support needed, length of stay and course in hospital, and in-hospital mortality. We previously documented high inter-rater agreement for chart abstraction on key variables by CCEDRRN research assistants [9].

Research assistants abstracted physician-assigned diagnoses from discharge summaries or consultation notes. They categorized primary and secondary discharge diagnoses according to a predefined dropdown menu of diagnoses that had been developed during the early pandemic, when recognized cases presented with acute respiratory syndromes. Research assistants documented all other discharge diagnoses using free text. Research assistants were unaware of the study purpose at the time of data abstraction.

Three pairs of physicians (CMH and one of FS/MS/DKT), all of whom had clinical experience treating COVID-19 patients, independently reviewed the medical records of 100 randomly selected cases with diagnoses from the pre-defined dropdown menu in duplicate. Physician assessments were blinded to the research assistants' notes. Physicians verified the research assistants' assigned primary symptom responsible for hospitalization (i.e., discharge diagnosis) and allocated these pre-defined dropdown menu diagnoses to hospitalizations primarily for COVID-19 versus with incidental SARS-CoV-2 infection (S2 Table). All other charts with these same pre-defined menu diagnoses were classified according to this allocation.

Two physicians with clinical experience treating COVID-19 (JT/LG) reviewed all free text diagnoses and assigned them to one of three categories: (a) primarily for COVID-19 if the free text diagnosis described a diagnosis listed in S3 Table (e.g., atypical pneumonia assigned to viral pneumonia), (b) with incidental SARS-CoV-2 if the diagnosis was clearly unrelated to COVID-19 (e.g., ectopic pregnancy), and (c) 'uncertain' if the diagnosis could have been COVID-19 related (e.g., fall, acute kidney injury, delirium; S4 Table). All discordant categorizations of free text diagnoses were categorized as uncertain. Three pairs of physicians (CMH and one of FS/MS/DKT) independently reviewed the medical records of all patients with uncertain diagnoses (S4 Table) and allocated them to admissions primarily for COVID-19 or with incidental SARS-CoV-2 infection. Initial disagreement between physician reviewers was resolved by discussion until consensus was reached.

Outcomes

The primary outcome was the proportion of hospitalizations primarily for COVID-19. Secondary outcomes were the differences in those classified as hospitalized for COVID-19 based on our clinical definition, the CDC algorithm, and the Massachusetts method and the impact of differences between these on length of hospital stay, number of hospitalizations in the study period, oxygen supplementation including mechanical ventilation, Intensive Care Unit (ICU) admissions, ICU length of stay, and in-hospital mortality.

Statistical analysis

We summarized the data with descriptive statistics appropriate for the data type and distribution. We compared groups using t-tests for means, Wilcoxon rank sum tests for medians, and chi-square tests for proportions. We measured the interrater agreement for the categorization of being hospitalized for COVID-19 versus with incidental SARS-CoV-2 infection using Fleiss' Kappa with 95% confidence intervals (CIs) [19]. Patients missing variable information in the chart were categorized to the not condition (e.g., non-smoker), as these data are usually only documented when the condition exists. A physician (EP) grouped similar free text diagnoses (e.g., intertrochanteric hip fracture and hip fracture) into broader categories for analysis. We developed multivariable logistic regression models for the outcomes of mechanical ventilation, ICU admission and in-hospital mortality, and reported adjusted odds ratios (ORs) and their 95% CIs. Predictor variables were chosen *a priori* based on their clinical importance and prior literature: age, sex, obesity, secondary immunodeficiency (malignant neoplasm, transplant recipient, or moderate/severe liver disease), admission primarily for COVID-19 (versus with incidental SARS-CoV-2 infection), subvariant dominance, illicit substance use, and vaccination status [6, 10, 12, 20]. We also included presenting hospital site as a fixed effect in all multivariable models to adjust for clustering. Age was modelled as a continuous linear (on the logit) predictor and scaled to estimates the odds ratio per decade. We calculated the number of doses of COVID-19 vaccines patients received seven days prior to their emergency department visit and dichotomized this variable (any dose versus none) in all models. Lack of vaccination was clearly documented in the chart and the rate of vaccination in BC during the study period was high, so we grouped those with unknown vaccination status into the vaccinated group and performed sensitivity analyses to determine the impact of this decision. Patients with missing data for other categorical variables were assigned, where possible, to the "not" condition.

To calculate the proportion of cases where the clinical definition differed from the CDC algorithm and Massachusetts method, we divided the number of cases in which the categorizations were discordant by the total number of cases. We calculated the sensitivity of the CDC algorithm and Massachusetts method separately by dividing all hospitalizations for COVID-19

identified by these definitions in agreement with the clinical definition by all hospitalizations for COVID-19 using the clinical definition. We calculated the specificity of the CDC algorithm and Massachusetts method separately by dividing patients who were hospitalized with incidental SARS-CoV-2 according to these definitions in agreement with the clinical definition over the same category from the clinical definition. To assess the impact of differences in classification between the two methods, we repeated the same models described above separately using the CDC algorithm and Massachusetts method. A cell size restriction policy prohibited us from reporting counts of less than five. We performed the data analysis with SAS 9.4 [21].

Ethics approval

The University of BC Clinical Research Ethics Board reviewed and approved the study protocol and waived the need for informed consent (H20-01015), allowing us to capture a complete sample. Authors had access to identifiable information during data collection (i.e., patient charts) but data were anonymized prior to analysis.

Results

Between December 19, 2021, and May 31, 2022, there were 42,505 patient visits to a participating Emergency Department, 6,383 (15.0%) where the patient tested positive for SARS-CoV-2 (Fig 1). Of 1,651 (25.8%; 1,651/5,983) patients requiring admission, 858 (52.0%; 858/1,651) were hospitalized for COVID-19 (Table 1). All others were deemed to have had incidental SARS-CoV-2 infections. The inter-rater agreement on the classification of being admitted for COVID-19 versus with incidental SARS-CoV-2 infection was 0.89 (95% CI: 0.83, 0.96) between research assistants and physicians (S5 Table). The interrater agreement on the classification of hospitalizations that were assigned an uncertain discharge diagnosis by the treating clinician in the medical record was 0.93 (95% CI 0.88–0.97) between physicians (S6 Table). Physicians categorized 39.0% (112/287) of uncertain hospitalizations as being for COVID-19. The probabilities of the most common uncertain discharge diagnoses being categorized for COVID-19 are shown in S1 Fig and S7 and S8 Tables. Characteristics of patients hospitalized for COVID-19 versus with incidental SARS-CoV-2 infection are shown in Table 1.

Patients hospitalized for COVID-19 required supplemental oxygen more commonly (52.5% vs 19.7%, $p<0.001$), spent more days in ICU (median 6.5 vs 4.0, $p = 0.001$), and had greater in-hospital mortality (12.6% vs 6.4%, $p<0.001$) compared to patients hospitalized with incidental SARS-CoV-2 (Table 2). Adjusted analyses indicated a greater odds of critical care admission (OR 1.86, 95% CI 1.31, 2.63) and in-hospital mortality (OR 1.49, 95% CI 1.03, 2.16) among patients hospitalized for COVID-19 (Fig 2 and S9 Table), though the impact on in-hospital mortality was no longer significant when 163 patients with unknown vaccination status were removed from the model (OR 1.46, 95% CI 0.99, 2.16).

The CDC algorithm had a sensitivity of 82.9% (711/858, 95% CI 80.3%, 85.4%) for identifying hospitalizations for COVID-19, and specificity of 98.1% (778/793, 95% CI 97.2%, 99.1%). The CDC algorithm commonly differed from the clinical definition when the primary discharge diagnoses were pneumonia, failure to thrive, or altered level of consciousness (S7 Table). While summary measures of health resource utilization were similar comparing the CDC-assigned cases with those identified using the clinical definition, the total number of hospital and ICU beds incurred differed (Table 2). Among admissions classified by clinical decision as primarily for COVID-19, the CDC algorithm underestimated intubations by 16.7% (6/36), re-hospitalizations by 18.9% (7/37), ICU hospitalizations by 10.0% (12/120), hospital days by 18.7% (1975/10,584), ICU days by 18.5% (301.5/1629), and in-hospital mortality by 12.0% (13/108) in our study cohort (Table 3). Replacing the clinical definition by the CDC algorithm

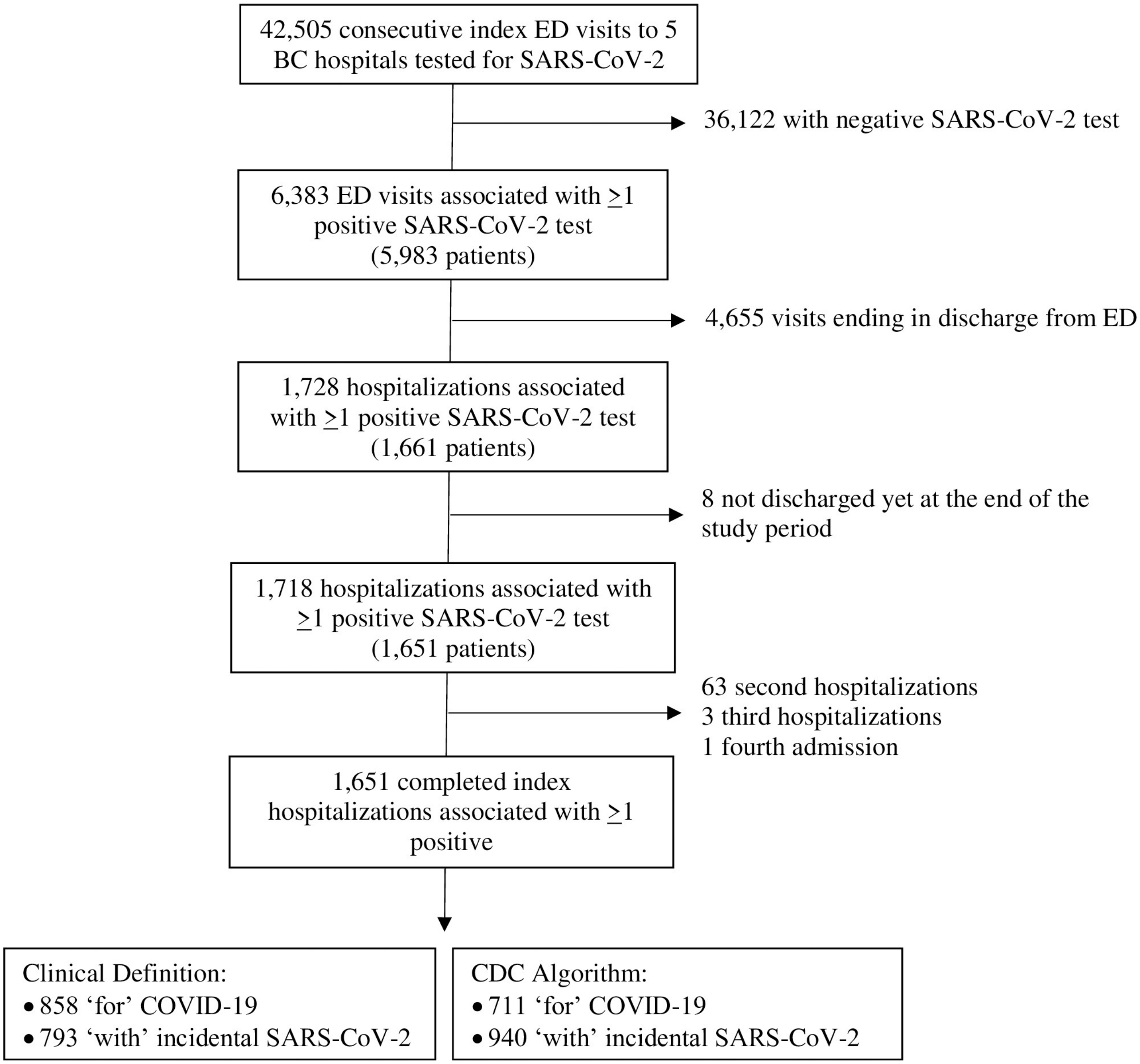

Fig 1. Flow diagram of enrolled patients. ED = emergency department; SARS-CoV-2 = Severe acute respiratory syndrome coronavirus 2.

https://doi.org/10.1371/journal.pone.0291580.g001

did not change the direction or statistical significance of the covariates in our regression models (Fig 2, S9 and S10 Tables). The same was true in sensitivity analyses where those with unknown vaccination status were removed.

The Massachusetts classification method had a sensitivity of 60.5% (519/858, 95% CI 57.2%, 63.8%) for identifying hospitalizations primarily for COVID-19, and a specificity of 78.6% (623/793, 95% CI 75.7%, 81.4%). By only taking into consideration the drugs administered to the patient, the Massachusetts method misclassified 229 cases with a primary discharge diagnosis of COVID-19 as incidental SARS-CoV-2, and 23 admissions for cancer as for COVID-19 (S8 Table). The prevalence of intubation, ICU admission, and in-hospital mortality was higher among admissions primarily for COVID-19 identified using the Massachusetts method compared to the clinical definition, but the incidence of these events was underestimated

Table 1. Patient and presentation characteristics by admission status using clinician decision.

	All Patients (n = 1,651)	Hospitalized primarily for COVID-19 (n = 858)	Hospitalized with incidental SARS-CoV-2 (n = 793)	P-value
**Omicron wave**[a]				
BA.1 dominant	1141 (69.1)	624 (72.7)	517 (65.2)	<0.001
Transition period	362 (21.9)	154 (18.0)	208 (26.2)	<0.001
BA.2 dominant	148 (9.0)	80 (9.3)	68 (8.6)	0.595
**Age in years (%)**				<0.001
<18	50 (3.0)	29 (3.4)	21 (2.7)	
18–24	42 (2.5)	9 (1.1)	33 (4.2)	
25–39	157 (9.5)	28 (3.3)	129 (16.3)	
40–64	453 (27.4)	214 (24.9)	239 (30.1)	
65–79	489 (29.6)	293 (34.2)	196 (24.7)	
>80	460 (27.9)	285 (33.2)	175 (22.1)	
**Male (%)**	987 (59.8)	536 (62.5)	451 (56.9)	0.021
**Arrival from (%)**				0.015
Home	1332 (80.7)	697 (81.2)	635 (80.1)	
Group-based settings (Long-term care, rehab facility, or corrections)	172 (10.4)	101 (11.8)	71 (9.0)	
Unstable housing[b]	97 (5.9)	39 (4.6)	58 (7.3)	
Inter-hospital transfer	38 (2.3)	18 (2.1)	20 (2.5)	
Other	12 (0.7)	Suppressed	9 (1.1)	
**Number of vaccine doses 7-days prior ED Visit (%)**				<0.001
Not vaccinated	286 (17.3)	193 (22.5)	93 (11.7)	
Vaccinated	1202 (72.8)	601 (70.1)	601 (75.8)	
One dose	63 (3.8)	31 (3.6)	32 (4.0)	
Two doses	223 (13.5)	108 (12.6)	115 (14.5)	
Three doses	246 (14.9)	144 (16.8)	102 (12.9)	
Unknown number of doses	670 (40.6)	318 (37.1)	352 (44.4)	
Unknown vaccination status	163 (9.9)	64 (7.5)	99 (12.5)	
**Most common comorbidities (%)**				
Hypertension	842 (51.0)	490 (57.1)	352 (44.4)	<0.001
Diabetes	519 (31.4)	302 (35.2)	217 (27.4)	<0.001
Coronary artery disease	277 (16.8)	168 (19.6)	109 (13.8)	0.002
Chronic lung disease, not asthma	248 (15.0)	166 (19.4)	82 (10.3)	<0.001
Congestive heart failure	225 (13.6)	136 (15.9)	89 (11.2)	0.006
Active cancer	185 (11.2)	113 (13.2)	72 (9.1)	0.009
Asthma	135 (8.2)	82 (9.6)	53 (6.7)	0.033
Transplant	110 (6.7)	89 (10.4)	21 (2.7)	<0.001
Obesity	79 (4.8)	48 (5.6)	31 (3.9)	0.109
Moderate/severe liver disease	39 (2.4)	14 (1.6)	25 (3.2)	0.042
**Past/current tobacco use (%)**	428 (25.9)	220 (25.6)	208 (26.2)	0.785
**Past/current illicit substance use (%)**	240 (14.5)	78 (9.1)	162 (20.4)	<0.001
**Arrival by ambulance (%)**	958 (58.0)	546 (63.6)	412 (52.0)	<0.001
**Canadian Triage Acuity Scale (CTAS, %)**				<0.001
CTAS 1 (Resuscitation)	102 (6.2)	56 (6.5)	46 (5.8)	
CTAS 2 (Emergent)	754 (45.7)	446 (52.0)	308 (38.8)	
CTAS 3 (Urgent)	758 (45.9)	345 (40.2)	413 (52.1)	
CTAS 4 (Less Urgent)	35 (2.1)	10 (1.2)	25 (3.2)	

(*Continued*)

Table 1. (Continued)

	All Patients (n = 1,651)	Hospitalized primarily for COVID-19 (n = 858)	Hospitalized with incidental SARS-CoV-2 (n = 793)	P-value
CTAS 5 (Non-Urgent)	Suppressed	Suppressed	0 (0.0)	
Missing	Suppressed	0 (0.0)	Suppressed	
**Hypoxic at ED arrival, (%)**[c]	194 (11.8)	150 (17.5)	44 (5.6)	<0.001
**Most Common COVID-19 Symptoms at ED arrival (%)**				
Cough	703 (42.6)	506 (59.0)	197 (24.8)	<0.001
Dyspnea	691 (41.9)	483 (56.3)	208 (26.2)	<0.001
Nausea/vomiting	472 (28.6)	206 (24.0)	266 (33.5)	<0.001
Altered consciousness	463 (28.0)	245 (28.6)	218 (27.5)	0.631
Fever	431 (26.1)	313 (36.5)	118 (14.9)	<0.001
Chest pain	307 (18.6)	172 (20.1)	135 (17.0)	0.115
Chills	269 (16.3)	180 (21.0)	89 (11.2)	<0.001
Diarrhea	213 (12.9)	147 (17.1)	66 (8.3)	<0.001
Myalgia	183 (11.1)	99 (11.5)	84 (10.6)	0.541
Headache	169 (10.2)	99 (11.5)	70 (8.8)	0.069
Sore Throat	149 (9.0)	105 (12.2)	44 (5.6)	<0.001
Weakness	54 (3.2)	42 (4.9)	12 (1.5)	<0.001
Dysgeusia/anosmia	22 (1.3)	17 (2.0)	5 (0.6)	0.017
**Symptom duration, median [IQR]**[d]	3.0 [1.0, 7.0]	4.0 [1.0, 7.0]	2.0 [0.0, 7.0]	<0.001
**Physiologic criteria for WHO severe disease in ED (%)**[e]	516 (31.3)	382 (44.5)	134 (16.9)	<0.001

ED = emergency department; IQR = [25th percentile, 75th percentile]; WHO = World Health Organization

[a] BA.1 dominant from December 19,2021 –February 12, 2022; BA.2 dominant from April 3, 2022 onwards

[b] Unstable housing includes homeless, shelter, single room occupancy

[c] We defined hypoxia as an arrival oxygen saturation below 92%.

[d] Missing for 219 patients

[e] We defined presentations for severe COVID-19 disease according to WHO age-based criteria. For adults, criteria for severe COVID-19 were met if the patient had an oxygen saturation of <90% on room air, a respiratory rate >30 breaths per minute, or signs of severe respiratory distress documented in the ED medical record [17].

Cell sizes less than five were suppressed

https://doi.org/10.1371/journal.pone.0291580.t001

throughout (Tables 2 and 3). The number of hospital and ICU bed days among admissions for COVID-19 were highest among the group classified by clinical decision, followed by the Massachusetts method, and finally the CDC algorithm (Table 2). Replacing the clinical definition by the Massachusetts method changed the significance or magnitude of some of the covariates in our regression models, notably removing the impact of vaccine effectiveness on mechanical ventilation and inflating the impact of being hospitalized primarily for COVID-19 on all measures of health services utilization (Fig 2, S9 and S11 Tables). When 163 patients with unknown vaccination status were removed from the models, we observed a protective association between vaccination and mechanical ventilation (OR 0.41, 95% CI 0.22–0.78) with no change in the association between vaccination and hospitalization primarily for COVID-19 (OR 5.15, 95% 2.47,10.75 versus OR 3.85, 95% 2.17, 6.85).

In our regressions, there were consistent findings no matter how admission for COVID-19 was defined (S9–S11 Tables). ICU admissions were more common among younger patients ($p \leq 0.001$) and death occurred more often among older patients ($p<0.0001$). Patients with a secondary immunodeficiency were more likely to die than those without ($p \leq 0.002$) and patients

Table 2. Resource utilization and outcomes of 1,651 patients, by admission status and classification method.

	All Patients (n = 1,651)	Clinical Decision			CDC Algorithm			Massachusetts Method		
		primarily for COVID-19 (n = 858)	with incidental SARS-CoV-2 (n = 793)	P-value	primarily for COVID-19 (n = 726)	with incidental SARS-CoV-2 (n = 925)	P-value	primarily for COVID-19 (n = 688)	with incidental SARS-CoV-2 (n = 960)	P-value
Supplemental oxygen (%)	606 (36.7)	450 (52.5)	156 (19.7)	<0.001	426 (58.7)	180 (19.5)	<0.001	465 (67.5)	141 (14.7)	<0.001
Oxygen delivery in ED (%)[a]				<0.001			<0.001			<0.001
Nasal prongs	400 (24.3)	291 (34.0)	109 (13.8)		278 (38.3)	122 (13.2)		297 (43.2)	103 (10.7)	
Simple or non-rebreather	62 (3.8)	51 (6.0)	11 (1.4)		45 (6.2)	17 (1.8)		52 (7.6)	10 (1.0)	
High-flow nasal oxygen	79 (4.8)	68 (7.9)	11 (1.4)		67 (9.2)	12 (1.3)		68 (9.9)	11 (1.2)	
CPAP/BiPAP	16 (1.0)	10 (1.2)	6 (0.8)		10 (1.4)	6 (0.7)		11 (1.6)	5 (0.5)	
Mechanical ventilation	67 (4.1)	36 (4.2)	31 (3.9)		30 (4.1)	37 (4.0)		46 (6.7)	21 (2.2)	
Hospitalizations				0.273			0.552			0.334
One admission (%)	1588 (96.2)	821 (95.7)	767 (96.7)		696 (95.9)	892 (96.4)		659 (95.7)	929 (95.6)	
≥2 hospitalizations (%)	63 (3.8)	37 (4.3)	26 (3.3)		30 (4.1)	33 (3.6)		30 (4.4)	33 (3.4)	
Index admission, days[b]										
Total, days	20368	10584	9784		8888	11480		9989	10379	
Median [IQR]	7.0 [3.0, 15.0]	7.0 [4.0, 14.0]	7.0 [3.0, 15.0]	0.348	7.0 [4.0, 14.0]	7.0 [3.0, 15.0]	0.283	9.0 [5.0, 17.0]	5.0 [3.0, 13.0]	<0.001
Mean (SD)	12.4 (15.7)	12.4 (15.8)	12.4 (15.7)	0.755	12.3 (15.7)	12.5 (15.8)	0.806	14.6 (16.3)	10.8 (15.2)	<0.001
Hospitalized to ICU (%)	185 (11.2)	120 (14.0)	65 (8.2)	<0.001	113 (15.6)	72 (7.8)	<0.001	136 (19.7)	49 (5.1)	<0.001
Index ICU admission, days										
Total, days	2055	1629	426		1345	710		1583	472	
Median [[IQR]	5.0 [2.0, 13.0]	6.5 [3.0, 16.0]	4.0 [1.0, 8.0]	0.001	6.0 [3.0, 14.0]	4.0 [1.0, 9.5]	0.020	6.0 [3.0, 14.0)	4.0 [2.0, 10.0]	0.052
Mean (SD)	11.1 (19.3)	13.6 (22.7)	6.6 (9.0)	<0.001	11.9 (18.5)	9.9 (20.6)	0.316	11.6 (18.6)	9.6 (21.2)	0.534
In-hospital mortality (%)	159 (9.6)	108 (12.6)	51 (6.4)	<0.001	101 (13.9)	58 (6.3)	<0.001	124 (18.0)	35 (3.6)	<0.001

ED = Emergency Department; IQR = [25th percentile, 75th percentile]; CPAP = Continuous Positive Airway Pressure; BiPAP = Bilevel Airway Pressure; ICU = intensive care unit

[a] 3 missing most aggressive form of oxygen delivery in ED excluded

[b] 6 missing discharge date excluded

https://doi.org/10.1371/journal.pone.0291580.t002

presenting during the Omicron BA.2 wave were less likely to die than those presenting during the Omicron BA.1 wave (p≤0.04). Patients who used illicit substances were more likely to be mechanically ventilated than those that did not (p≤0.04). When admissions were classified using the clinical definition or the CDC algorithm, odds of mechanical ventilation, critical care admission, and mortality were all significantly higher among males than females (S9 and S10 Tables). Obesity had no impact of odds of mechanical ventilation, ICU admission or death irrespective of the classification system used.

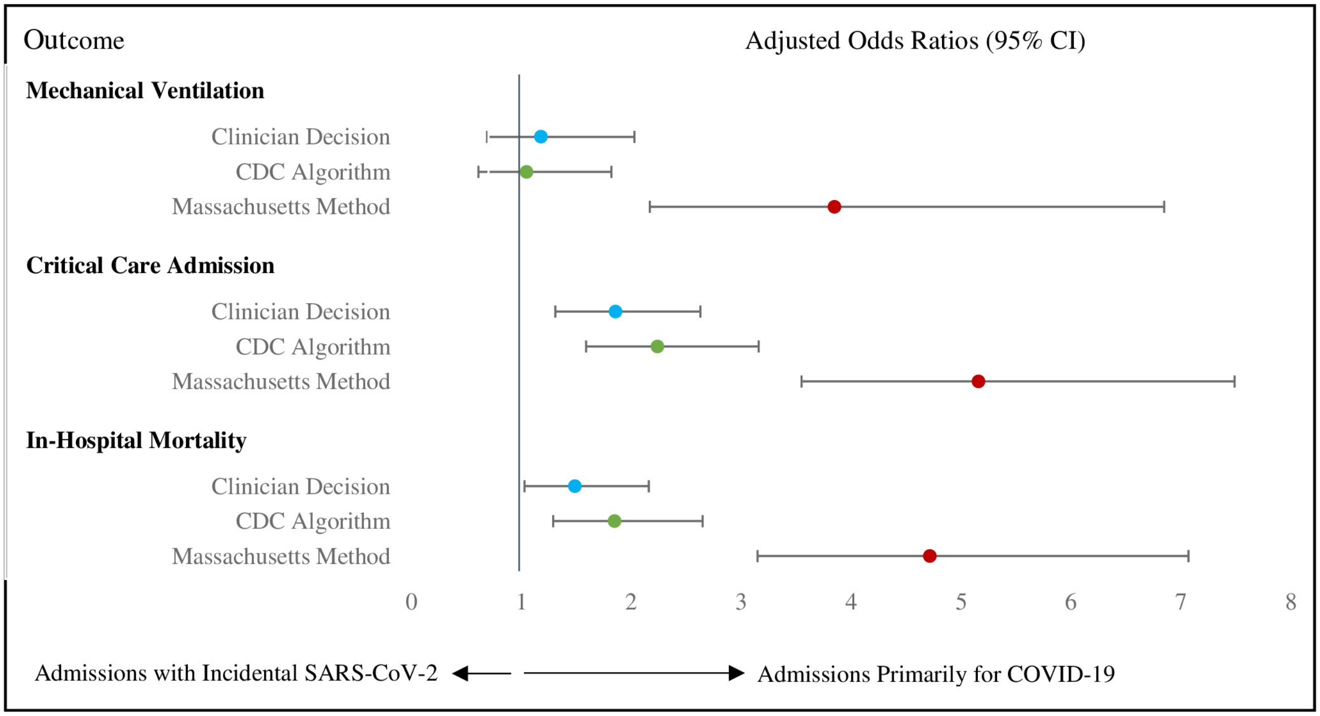

Fig 2. Adjusted odds of ventilation, critical care admission or mortality among 1,651 SARS-CoV-2 positive patients who were hospitalized primarily for COVID-19 versus with incidental SARS-CoV-2 determined using either clinician decision, the CDC algorithm or the Massachusetts methods. CI = confidence interval. Models for each outcome were adjusted for age, sex, presenting hospital, secondary immunodeficiency (i.e., active malignant neoplasm, transplant recipient, moderate/severe liver disease), obesity, Omicron subvariant, illicit substance use, and COVID-19 vaccinations. The reference standard is hospitalized with incidental SARS-CoV-2. Adjusted odds ratios and 95% confidence intervals for factors included in each regression are presented in S9–S11 Tables (blue circle = S9 Table, green circle = S10 Table, red circle = S11 Table).

https://doi.org/10.1371/journal.pone.0291580.g002

Discussion

As we transition to endemic COVID-19, continued public health surveillance, research, and planning are needed to ensure evaluation of and planning for future waves of infection as the virus, population-level immunity, and other health system pressures evolve. Population-level administrative health data linked with SARS-CoV-testing data have facilitated research and surveillance thus far, but have not taken into account the substantial proportion of asymptomatic or minimally symptomatic infections identified among hospitalized patients: We found that only half of all SARS-CoV-2 positive patients admitted to hospital during the Omicron wave were admitted because of COVID-19, with substantially different health resource utilization between those admitted for COVID-19 compared to those admitted with incidental SARS-CoV-2 infections. Misclassifying admissions substantially impacts health resource utilization estimates.

We found lower rates of hospitalizations for COVID-19 during the Omicron wave compared to those reported in prior waves [6, 22–24]. Half of SARS-CoV-2 positive hospitalized patients were hospitalized with incidental SARS-CoV-2 throughout the BA.1 and BA.2 dominant periods. Patients hospitalized for COVID-19 incurred more critical care resources and died more frequently compared to those hospitalized with incidental infection. Interestingly, the overall mortality of 9.6% among patients in our study was comparable to that found in other studies on Omicron that did not stratify hospitalizations into hospitalizations for COVID-19 versus with incidental SARS-CoV-2 [25]. Our results illustrate how reporting overall mortality rates among hospitalized SARS-CoV-2 patients has the potential to underestimate

Table 3. Resource utilization and outcomes of 858 patients hospitalized primarily for COVID-19 using clinician decision, identified by the CDC algorithm or Massachusetts method.

	Clinical Decision Primarily for COVID-19 (n = 858)	CDC Primarily for COVID-19 (n = 711)	Massachusetts Primarily for COVID-19 (n = 519)
Supplemental oxygen (%)	450 (52.5)	418 (58.8)	387 (74.6)
Most aggressive form of oxygen delivery in ED (%)[a]			
Nasal prongs	291 (34.0)	271 (38.2)	246 (47.5)
Simple or non-rebreather facemask	51 (6.0)	45 (6.3)	46 (8.9)
High-flow nasal oxygen	68 (7.9)	66 (9.3)	61 (11.8)
CPAP/BiPAP	10 (1.2)	10 (1.4)	7 (1.4)
Mechanical ventilation	36 (4.2)	30 (4.2)	32 (6.2)
Hospitalizations			
One admission (%)	821 (95.7)	681 (95.8)	497 (95.8)
Two or more hospitalizations (%)	37 (4.3)	30 (4.2)	22 (4.2)
Hospital days in first admission[b]			
Total hospital days	10584	8,609	7,452
Mean length of stay (SD)	12.4 (15.8)	12.2 (15.5)	14.4 (16.4)
Median hospital days [IQR]	7.0 [4.0, 14.0]	7.0 [4.0, 14.0]	9.0 [5.0, 17.0]
Hospitalized to ICU (%)	120 (14.0)	108 (15.2)	109 (21.0)
Critical care days during first admission			
Total critical care days	1629	1327.5	1388.5
Mean length of stay (SD)	13.6 (22.7)	12.3 (18.8)	12.7 (20.2)
Median critical care days [IQR]	6.5 [3.0, 16.0]	6.0 [3.0, 15.0]	6.0[3.0, 16.0]
In-hospital mortality (%)	108 (12.6)	95 (13.4)	91 (17.5)

ED = Emergency Department; IQR = [25th percentile, 75th percentile]; CPAP = Continuous Positive Airway Pressure; BiPAP = Bilevel Airway Pressure; ICU = intensive care unit

[a] 3 missing most aggressive form of oxygen delivery in ED

[b] 6 missing discharge date

https://doi.org/10.1371/journal.pone.0291580.t003

mortality for patients hospitalized for COVID-19 (12.6%), who had substantially higher mortality compared to patients with incidental SARS-CoV-2 (6.4%) which could lead to underestimation of virulence in future waves [26]. Given incomplete community-based case ascertainment, correct stratification of hospitalizations is needed to ensure accurate estimation of in-hospital mortality for COVID-19. This will be critical to understanding the virulence of future variants and in guiding public health decision-making.

Our study found disagreements between diagnoses assigned by experienced COVID-19 clinicians and a diagnosis-based algorithm defined by the CDC in 10% of cases, and with the Massachusetts method in almost 40% of cases. This level of misclassification resulted in an 18.6% underestimation of COVID-19 attributable hospital *and* ICU bed day utilization by the CDC algorithm, and a 27.6% underestimation by the Massachusetts methods. This level of misclassification could have a substantive negative impact on health resource planning if applied for future waves. Applying our estimates to Canadian health data reported on October 31, 2022, use of the CDC algorithm would have underestimated hospital resources being used to look after current COVID-19 hospitalizations by 14,425 ward bed-days and 1,432 ICU bed-days across the country [27]. In a health care system that is already under tremendous strain and as SARS-CoV-2 continues to mutate and spread, more accurate resource utilization estimations are desirable.

The specificity of the CDC-based algorithm was high, indicating that the CDC algorithm can likely be used in administrative records in study designs that require high specificity, such

Criteria for identifying COVID-19 hospitalizations as primarily for COVID-19*:

1) a U07.1 primary discharge diagnosis OR
2) a U07.1 secondary discharge diagnosis AND:
    a. treatment with remdesivir OR
    b. treatment with dexamethasone OR
    c. a primary discharge diagnosis of sepsis, pulmonary embolism, acute respiratory failure, or pneumonia

Fig 3. Potential adjustment to the CDC algorithm suggested by Adjei et al. to improve sensitivity for resource intensive cases for the purposes of health care resource planning [6]. *Text taken directly from Adjei et al. with the exception of the italicized addition.

https://doi.org/10.1371/journal.pone.0291580.g003

as for vaccine effectiveness estimation [28]. However, sensitivity was limited, with the CDC-based algorithm missing close to one in five hospitalizations for COVID-19. Modelling developed using the CDC definition will likely underestimate health resource utilization.

The Massachusetts state department's method of using dexamethasone administration alone as an indicator of hospitalization primarily for COVID-19 had both lower sensitivity and specificity than the CDC algorithm. This is not surprising, as dexamethasone is only indicated for moderate-to-severe COVID-19 [3]. As a result, the use of dexamethasone administration to identify COVID-19 admissions in administrative data missed 40% of hospitalizations identified by clinicians as having been primarily for COVID-19. As dexamethasone is not exclusively used to treated COVID-19, it is not surprising that the Massachusetts methods was non-specific.

When estimating the burden of COVID-19 on the health care system, it is possible that adding dexamethasone administration to the CDC algorithm alongside remdesivir may help improve the performance of the CDC definition, in particular for cases that required supplemental oxygen and respiratory support who may not have received remdesivir (Fig 3). Diagnoses such as hyponatremia, acute kidney injury or failure to thrive, felt to be COVID-19 related by clinicians, that were not identified by the CDC discharge diagnosis-based algorithm, would likely continue to be missed. Interestingly, the CDC algorithm incorrectly attributed many sepsis cases, for example cases of bacteremia, to COVID-19. Future studies should focus on uncertain diagnoses, to understand how these hospitalizations could more accurately be classified by considering other discharge diagnoses, treatments, and diagnostic tests. Alternatively, algorithmically assigned diagnoses such as sepsis, could be weighted by their likelihood of being attributable to COVID-19 versus other causes if future studies corroborate our findings.

Our study was not without limitations. As we used retrospectively collected data, our assessments were limited to what was documented in records. We were unable to obtain individual-level genomic sequencing data, and assigned the sub-variant based on the patient's admission date. As a result, we were unable to allocate patients to a subvariant during the transition period between BA.1 and BA.2, and had few BA.2 patients. Small cells sizes for in-hospital mortality, Omicron BA.2, and illicit substance use resulted in loss of power during sensitivity analyses when 163 patients with unknown vaccination status were removed from our regression models. We were unable to recruit patients in rural settings or outside of British Columbia which limits the generalizability of our results. Lastly, while no criterion standard currently exists for the diagnosis of being hospitalized primarily for COVID-19, we were able to demonstrate excellent inter-rater agreement during each phase of our chart review process, and therefore believe that the definitions we propose are robust.

Conclusion

In this multi-center study during the Omicron wave, half of hospitalizations among SARS--CoV-2 positive patients were primarily for COVID-19, and associated with greater risk of poor health outcomes, and more hospital resource utilization compared to hospitalizations with incidental SARS-CoV-2 infection. Hospital resource utilization was underestimated substantially by the CDC algorithm; future studies should investigate the impact of adding dexamethasone prescription to the CDC algorithm to better isolate SARS-CoV-2 positive patients hospitalized primarily for COVID-19.

Supporting information

S1 File. Contributors to the Canadian COVID-19 Emergency Department Rapid Response Network.
(DOCX)

S1 Fig. Probability of most common uncertain discharge diagnoses being categorized primarily for COVID-19. The x-axis depicts the probability of being adjudicated by clinicians as an admission primarily for COVID-19 among all cases with the same primary discharge diagnosis that were abstracted from the medical record.
(DOCX)

S1 Table. Hospital sites and dates of consecutive data entry.
(DOCX)

S2 Table. Predefined drop-down menu of COVID-19 related diagnoses allocated to the hospitalized primarily for COVID-19 category.
(DOCX)

S3 Table. Free text diagnoses allocated to the hospitalized primarily for COVID-19 category.
(DOCX)

S4 Table. Free text discharge diagnoses deemed 'uncertain' if with incidental COVID-19 or 'for' incidental COVID-19.
(DOCX)

S5 Table. Interrater agreement for classifying patients hospitalized primarily for COVID-19 and with incidental SARS-CoV-2 between research assistants and two physicians.
(DOCX)

S6 Table. Interrater agreement for classifying patients hospitalized primarily for COVID-19 and with incidental SARS-CoV-2 who had a free text diagnosis deemed to be uncertain.
(DOCX)

S7 Table. The most common primary discharge diagnoses among discordant cases when comparing the clinical decision and the CDC methods of classification.
(DOCX)

S8 Table. The most common primary discharge diagnoses among discordant cases when comparing the clinical decision and the Massachusetts method of classification.
(DOCX)

S9 Table. Factors associated with ventilation, critical care admission or mortality among 1,651 SARS-CoV-2 positive patients, according to clinician decision. OR = odds ratio;

ICU = intensive care unit; CI = confidence interval. [a] active malignant neoplasm, transplant recipient, moderate/severe liver disease. Hospital site was included as a fixed effect in this model. For simplicity, site estimates were excluded from the table.
(DOCX)

S10 Table. Factors associated with ventilation, critical care admission or mortality among 1,651 SARS-CoV-2 positive patients, according to the CDC admission classification.
OR = odds ratio; ICU = intensive care unit; CI = confidence interval. [a] active malignant neoplasm, transplant recipient, moderate/severe liver disease. Hospital site was included as a fixed effect in this model. For simplicity, site estimates were excluded from the table.
(DOCX)

S11 Table. Factors associated with ventilation, critical care admission or mortality among 1,651 SARS-CoV-2 positive patients, according to the Massachusetts admission classification. OR = odds ratio; ICU = intensive care unit; CI = confidence interval. [a] active malignant neoplasm, transplant recipient, moderate/severe liver disease. Hospital site was included as a fixed effect in this model. For simplicity, site estimates were excluded from the table.
(DOCX)

Author Contributions

Conceptualization: Corinne M. Hohl, Amber Cragg, Elizabeth Purssel, Finlay A. McAlister, Daniel K. Ting, Frank Scheuermeyer, Maja Stachura, Lars Grant, John Taylor, Ivy Cheng, Clare L. Atzema, Rajan Bola, Laurie J. Morrison, Megan Landes, Jeffrey J. Perry, Rhonda J. Rosychuk.

Data curation: Jeffrey P. Hau, Rhonda J. Rosychuk.

Formal analysis: Amber Cragg, Rajan Bola.

Funding acquisition: Corinne M. Hohl, Daniel K. Ting.

Investigation: Corinne M. Hohl, Daniel K. Ting, Frank Scheuermeyer, Maja Stachura, Lars Grant, John Taylor, Ivy Cheng, Megan Landes, Jeffrey J. Perry.

Methodology: Corinne M. Hohl, Amber Cragg, Elizabeth Purssel, Finlay A. McAlister, Daniel K. Ting, Frank Scheuermeyer, Maja Stachura, Lars Grant, John Taylor, Ivy Cheng, Clare L. Atzema, Rajan Bola, Laurie J. Morrison, Megan Landes, Jeffrey J. Perry, Rhonda J. Rosychuk.

Project administration: Amber Cragg, Josephine Kanu, Jeffrey P. Hau, Rajan Bola.

Resources: Corinne M. Hohl.

Supervision: Corinne M. Hohl, Finlay A. McAlister, Jeffrey P. Hau, Ivy Cheng, Laurie J. Morrison, Rhonda J. Rosychuk.

Validation: Corinne M. Hohl, Amber Cragg, Elizabeth Purssel, Finlay A. McAlister, Daniel K. Ting, Frank Scheuermeyer, Maja Stachura, Lars Grant, John Taylor, Jeffrey P. Hau, Rajan Bola, Rhonda J. Rosychuk.

Visualization: Amber Cragg, Rajan Bola.

Writing – original draft: Corinne M. Hohl, Amber Cragg, Daniel K. Ting, Rajan Bola.

Writing – review & editing: Corinne M. Hohl, Amber Cragg, Elizabeth Purssel, Finlay A. McAlister, Daniel K. Ting, Frank Scheuermeyer, Maja Stachura, Lars Grant, John Taylor,

Josephine Kanu, Jeffrey P. Hau, Ivy Cheng, Clare L. Atzema, Laurie J. Morrison, Megan Landes, Jeffrey J. Perry, Rhonda J. Rosychuk.

References

1. Murray CJL. COVID-19 will continue but the end of the pandemic is near. The Lancet. 2022; 399: 417–419. https://doi.org/10.1016/S0140-6736(22)00100-3 PMID: 35065006
2. Health Canada. COVID-19 wastewater surveillance dashboard. 27 Sep 2022 [cited 3 Oct 2022]. Available: https://health-infobase.canada.ca/covid-19/wastewater/
3. Fillmore NR, La J, Zheng C, Doron S, Do NV, Monach PA, et al. The COVID-19 hospitalization metric in the pre- and postvaccination eras as a measure of pandemic severity: A retrospective, nationwide cohort study. Infect Control Hosp Epidemiol. 2022; 1–6. https://doi.org/10.1017/ice.2022.13 PMID: 35012694
4. Català M, Coma E, Alonso S, Andrés C, Blanco I, Antón A, et al. Transmissibility, hospitalization, and intensive care admissions due to omicron compared to delta variants of SARS-CoV-2 in Catalonia: A cohort study and ecological analysis. Front Public Health. 2022; 10: 961030. https://doi.org/10.3389/fpubh.2022.961030 PMID: 36033822
5. Menni C, Valdes AM, Polidori L, Antonelli M, Penamakuri S, Nogal A, et al. Symptom prevalence, duration, and risk of hospital admission in individuals infected with SARS-CoV-2 during periods of omicron and delta variant dominance: a prospective observational study from the ZOE COVID Study. Lancet. 2022; 399: 1618–1624. https://doi.org/10.1016/S0140-6736(22)00327-0 PMID: 35397851
6. Adjei S, Hong K, Molinari N-AM, Bull-Otterson L, Ajani UA, Gundlapalli AV, et al. Mortality Risk Among Patients Hospitalized Primarily for COVID-19 During the Omicron and Delta Variant Pandemic Periods—United States, April 2020–June 2022. MMWR Morb Mortal Wkly Rep. 2022; 71: 1182–1189. https://doi.org/10.15585/mmwr.mm7137a4 PMID: 36107788
7. Habas E, Ali E, Habas A, Rayani A, Ghazouani H, Khan F, et al. Hyponatremia and SARS-CoV-2 infection: A narrative review. Medicine (Baltimore). 2022; 101: e30061. https://doi.org/10.1097/MD.0000000000030061 PMID: 35960124
8. Fatima S. Here's why the state is changing the way it reports COVID hospitalization data—The Boston Globe. In: BostonGlobe.com [Internet]. 07 2022 [cited 8 Feb 2023]. Available: https://www.bostonglobe.com/2022/01/07/nation/heres-why-state-is-changing-way-it-reports-covid-hospitalization-data/
9. Hohl CM, Rosychuk RJ, McRae AD, Brooks SC, Archambault P, Fok PT, et al. Development of the Canadian COVID-19 Emergency Department Rapid Response Network population-based registry: a methodology study. CMAJ Open. 2021; 9: E261–E270. https://doi.org/10.9778/cmajo.20200290 PMID: 33731427
10. Hohl CM, Rosychuk RJ, Hau JP, Hayward J, Landes M, Yan JW, et al. Treatments, Resource Utilization, and Outcomes of COVID-19 Patients Presenting to the Emergency Department across Pandemic Waves. Can J Emerg Med. 2022 [cited 23 Nov 2021]. https://doi.org/10.1101/2021.07.30.21261288
11. McRae AD, Hohl CM, Rosychuk R, Vatanpour S, Ghaderi G, Archambault PM, et al. CCEDRRN COVID-19 Infection Score (CCIS): development and validation in a Canadian cohort of a clinical risk score to predict SARS-CoV-2 infection in patients presenting to the emergency department with suspected COVID-19. BMJ Open. 2021; 11: e055832. https://doi.org/10.1136/bmjopen-2021-055832 PMID: 34857584
12. Hohl CM, Rosychuk RJ, Archambault PM, O'Sullivan F, Leeies M, Mercier É, et al. The CCEDRRN COVID-19 Mortality Score to predict death among nonpalliative patients with COVID-19 presenting to emergency departments: a derivation and validation study. CMAJ Open. 2022; 10: E90–E99. https://doi.org/10.9778/cmajo.20210243 PMID: 35135824
13. Archived B.C. COVID-19 Data. [cited 21 Jul 2023]. Available: http://www.bccdc.ca/health-info/diseases-conditions/covid-19/archived-b-c-covid-19-data#variants
14. Elimian KO, Ochu CL, Ebhodaghe B, Myles P, Crawford EE, Igumbor E, et al. Patient characteristics associated with COVID-19 positivity and fatality in Nigeria: retrospective cohort study. BMJ Open. 2020; 10: e044079. https://doi.org/10.1136/bmjopen-2020-044079 PMID: 33334842
15. Hohl CM, Hau JP, Vaillancourt S, Grant J, Brooks SC, Morrison LJ, et al. Sensitivity and Diagnostic Yield of the First SARS-CoV-2 Nucleic Acid Amplification Test Performed for Patients Presenting to the Hospital. JAMA Netw Open. 2022; 5: e2236288. https://doi.org/10.1001/jamanetworkopen.2022.36288 PMID: 36223119
16. WHO global clinical platform for COVID-19: core case report form (CRF), version 8 April 2020, revised 13 July 2020, revised 29 November 2021. [cited 16 Dec 2022]. Available: https://www.who.int/publications-detail-redirect/WHO-2019-nCoV-Clinical-CRF-2021.1

17. World Health Organization. COVID-19 Clinical management: living guidance. 21 Jan 2021. Available: https://www.who.int/publications/i/item/WHO-2019-nCoV-clinical-2023.1

18. Mirhaghi A, Heydari A, Mazlom R, Ebrahimi M. The Reliability of the Canadian Triage and Acuity Scale: Meta-analysis. North Am J Med Sci. 2015; 7: 299–305. https://doi.org/10.4103/1947-2714.161243 PMID: 26258076

19. Fleiss JL. Measuring nominal scale agreement among many raters. Psychol Bull. 1971; 76: 378–382. https://doi.org/10.1037/h0031619

20. Brooks SC, Rosychuk RJ, Perry JJ, Morrison LJ, Wiemer H, Fok P, et al. Derivation and validation of a clinical decision rule to risk-stratify COVID-19 patients discharged from the emergency department: The CCEDRRN COVID discharge score. J Am Coll Emerg Physicians Open. 2022;3. https://doi.org/10.1002/emp2.12868 PMID: 36579029

21. SAS/ACCESS(r) 9.4 Interface to ADABAS. Cary, NC: SAS Institute Inc 2013.

22. Abdullah F, Myers J, Basu D, Tintinger G, Ueckermann V, Mathebula M, et al. Decreased severity of disease during the first global omicron variant covid-19 outbreak in a large hospital in tshwane, south africa. Int J Infect Dis. 2022; 116: 38–42. https://doi.org/10.1016/j.ijid.2021.12.357 PMID: 34971823

23. Antwi-Amoabeng D, Beutler BD, Chahal G, Mahboob S, Gullapalli N, Tedja R, et al. Ensuring trust in COVID-19 data: A retrospective cohort study. Medicine (Baltimore). 2021; 100: e26972. https://doi.org/10.1097/MD.0000000000026972 PMID: 34477127

24. Voor in 't holt AF, Haanappel CP, Rahamat–Langendoen J, Molenkamp R, van Nood E, van den Toorn LM, et al. Admissions to a large tertiary care hospital and Omicron BA.1 and BA.2 SARS-CoV-2 polymerase chain reaction positivity: primary, contributing, or incidental COVID-19. Int J Infect Dis. 2022; 122: 665–668. https://doi.org/10.1016/j.ijid.2022.07.030 PMID: 35842214

25. Bouzid D, Visseaux B, Kassasseya C, Daoud A, Fémy F, Hermand C, et al. Comparison of Patients Infected With Delta Versus Omicron COVID-19 Variants Presenting to Paris Emergency Departments: A Retrospective Cohort Study. Ann Intern Med. 2022; 175: 831–837. https://doi.org/10.7326/M22-0308 PMID: 35286147

26. McAlister FA, Hau JP, Atzema C, McRae AD, Morrison LJ, Grant L, et al. The burden of incidental SARS-CoV-2 infections in hospitalized patients across pandemic waves in Canada. Sci Rep. 2023; 13: 6635. https://doi.org/10.1038/s41598-023-33569-2 PMID: 37095174

27. Government of Canada. COVID-19 daily epidemiology update—Canada.ca. [cited 16 Jan 2023]. Available: https://health-infobase.canada.ca/covid-19/epidemiological-summary-covid-19-cases.html#VOC

28. De Serres G, Skowronski DM, Wu XW, Ambrose CS. The test-negative design: validity, accuracy and precision of vaccine efficacy estimates compared to the gold standard of randomised placebo-controlled clinical trials. Eurosurveillance. 2013;18. https://doi.org/10.2807/1560-7917.ES2013.18.37.20585 PMID: 24079398
